# Additive Effects of Cyclic Peptide [R4W4] When Added Alongside Azithromycin and Rifampicin against *Mycobacterium avium* Infection

**DOI:** 10.3390/pathogens12081057

**Published:** 2023-08-18

**Authors:** Melissa Kelley, Kayvan Sasaninia, Arbi Abnousian, Ali Badaoui, James Owens, Abrianna Beever, Nala Kachour, Rakesh Kumar Tiwari, Vishwanath Venketaraman

**Affiliations:** 1 Graduate College of Biomedical Sciences, Western University of Health Sciences, Pomona, CA 91766, USA; 2 College of Osteopathic Medicine of the Pacific, Western University of Health Sciences, Pomona, CA 91766, USA; ksasanina@westernu.edu (K.S.); arbi.abnousian@westernu.edu (A.A.); jamesowens@westernu.edu (J.O.); 3 College of Osteopathic Medicine, Kansas City University, Kansas City, MO 64106, USA; 4 School of Medicine, University of California Riverside, Riverside, CA 92521, USA; nkach001@ucr.edu; 5 Department of Biomedical and Pharmaceutical Sciences, Center for Targeted Drug Delivery, Chapman University School of Pharmacy, Harry and Diane Rinker Health Science Campus, Irvine, CA 92866, USA

**Keywords:** nontuberculous mycobacteria, *Mycobacterium avium*, azithromycin, rifampicin, immunocompromised

## Abstract

*Mycobacterium avium* (*M. avium*), a type of nontuberculous mycobacteria (NTM), poses a risk for pulmonary infections and disseminated infections in immunocompromised individuals. Conventional treatment consists of a 12-month regimen of the first-line antibiotics rifampicin and azithromycin. However, the treatment duration and low antibiotic tolerability present challenges in the treatment of *M. avium* infection. Furthermore, the emergence of multidrug-resistant mycobacterium strains prompts a need for novel treatments against *M. avium* infection. This study aims to test the efficacy of a novel antimicrobial peptide, cyclic [R4W4], alongside the first-line antibiotics azithromycin and rifampicin in reducing *M. avium* survival. Colony-forming unit (CFU) counts were assessed after treating *M. avium* cultures with varying concentrations of cyclic [R4W4] alone or in conjunction with azithromycin or rifampicin 3 h and 4 days post-treatment. *M. avium* growth was significantly reduced 4 days after cyclic [R4W4] single treatment. Additionally, cyclic [R4W4]–azithromycin and cyclic [R4W4]–rifampicin combination treatments at specific concentrations significantly reduced *M. avium* survival 3 h and 4 days post-treatment compared with single antibiotic treatment alone. These findings demonstrate cyclic [R4W4] as a potent treatment method against *M. avium* and provide insight into novel therapeutic approaches against mycobacterium infections.

## 1. Introduction

In recent decades, the tuberculosis (TB) incidence rate has been declining, while the incidence and prevalence of nontuberculous mycobacteria (NTM) infections have seen a rise in most areas of the world [1]. *Mycobacterium avium* complex (MAC) is one of the most common nontuberculous mycobacteria (NTM) contributing to pulmonary disease worldwide [2]. MAC is found ubiquitously in the environment, including soil, various water sources, animals (domestic and wild), as well as milk and food products [3]. The main reservoir for *M. avium* subsp. *avium* is found to be the environment, and only occasionally in mammals [4]. In contrast, the main reservoir for *M. avium* subsp. *paratuberculosis* is animals, which help to spread the infection through the fecal contamination of milk or the environment [5]. Through these reservoirs, *M. avium* subsp. *paratuberculosis* can eventually spread to humans via horizontal transmission, through direct contact or by utilizing the enteric route, or via vertical transmission, which happens through reproduction [5]. 

*M. avium* initially colonizes the respiratory or intestinal mucosa by evading protective barriers and infection of phagocytic cells. Monocytes and macrophages are the primary host cells of *M. avium*, and once the bacterium gains access to intracellular compartments, it utilizes mechanisms, such as the inhibition of phagosome–lysosome fusion, inhibition of acidification of the phagocytic vacuole, and other evasion techniques, to survive and proliferate [6,7]. The immune response against *M. avium* involves both the innate and adaptive immune systems. The innate immune response involves natural killer (NK) cells and the adaptive immune response is dependent on CD4 T cells [8,9]. However, in both cases, macrophages lead to the activation and proliferation of NK cells and T cells [10].

MAC causes pulmonary infections, disseminated infections, and lymphadenitis. Clinical phenotypes of pulmonary MAC infection include fibrocavitary disease and nodular bronchiectatic disease. Fibrocavitary disease forms focal or cavitary lesions found in the superior areas of the lungs. Nodular bronchiectatic disease forms bronchiectasis and small nodules in the middle lobes of the lungs. In accordance with the guidelines, MAC infection diagnosis requires the presence of pulmonary and systemic symptoms, a chest X-ray showing nodules/cavities or a CT scan showing bronchiectasis with nodules, and a lab culture of sputum from the lungs [11].

Patients with preexisting respiratory conditions have greater susceptibility to infection. Lung disease, such as chronic obstructive pulmonary disease (COPD), contributes to increased risk of MAC colonization [12,13]. COPD is an inflammatory condition that leads to the obstruction of airflow within the lungs. Smoking and the inhalation of toxins in air, the main causes of COPD, result in oxidative overload, since the lung’s antioxidative capacity cannot keep up [14]. Over longer periods of time, the chronic exposure to harmful toxins causes damage to the lung tissue, leaving patients’ immune systems vulnerable to infection.

MAC infection includes three major species that are acid-fast slow-growing mycobacteria: *M. avium*, *Mycobacterium intracellulare*, and *Mycobacterium chimaera*. They are difficult to distinguish, cause similar symptoms, and show some resistance to antibiotics [15]. MAC subtypes are associated with infections affecting the gastrointestinal, autoimmune, soft tissue, musculoskeletal, and other organ systems [16].

Pulmonary disease due to MAC infection is common in both immunocompetent and immunocompromised patients [16]. Immunocompromised patients, such as those with human immunodeficiency virus (HIV), severe combined immunodeficiency disease (SCID), or immune system cancers, which include leukemia and lymphoma, are at an increased risk of disseminated MAC infection [15,17]. In immunocompromised patients, mycobacterium can enter the lymphatic system and infect organs external to pulmonary locations [18]. Additionally, patients with immune-mediated inflammatory disease receiving corticosteroids, tumor necrosis factor (TNF)-α treatment, or TNF-α inhibitors, and organ transplant recipients taking immunosuppressive medications, are at a greater risk of NTM infections.

In the case of HIV, almost half of the patients with severely immunocompromised acquired immunodeficiency syndrome (AIDS) disease had disseminated MAC infections prior to antiviral therapy [19]. MAC infection poses a threat to HIV patients who do not have access to antiviral therapy or to those patients in which antiviral therapy is ineffective. HIV attacks the immune system, preferentially by infecting the CD4 T cells. Since CD4 T cells play an important role in signaling other immune cells, an individual with a lower CD4 cell count does not produce an adequate immune response, making HIV patients susceptible to MAC infections [3].

Antibiotic treatment for NTM infections could be a difficult choice due to adverse reactions to antibiotics and inconsistent disease progression. However, the progression of the infection can be predicted by factors such as cavitary lesions, a low body mass index, and poor nutrition. The progression of the infection is important, as diagnosis of MAC pulmonary disease does not always result in the initiation of treatment [20]. When the initiation of treatment is indicated, management for NTM infections, including MAC, involves a macrolide combination of azithromycin, rifampicin, and ethambutol for at least 12 months after detection [21,22]. Azithromycin is preferred over clarithromycin for better tolerance and drug interactions [23]. It should be noted that the treatment duration could lead to increased risk of ototoxicity and hepatotoxicity [24]. Specifically, patients with HIV are at an increased risk of disseminated MAC infection and are recommended to receive primary prophylaxis, especially in developing countries [25]. However, patients with access to highly active antiretroviral therapy (HAART) can forgo primary prophylaxis [26].

The frequency of treatment is also variable depending on the type of disease and patient compliance. In some cases of pulmonary disease in patients with MAC, a daily treatment regimen is recommended [27]. However, daily therapy increases the chances of adverse effects, such as visual disturbance [28]. In contrast, intermittent therapy for MAC infection shows a high success rate, low chances of adverse effects, higher tolerability, and higher patient compliance [29]. Intermittent therapy involves the administration of treatment three times a week in comparison with daily therapy, and usually lower doses, in some cases. Despite the improvement in treatment outcomes with a three-drug combination regimen, the long duration and adverse effects remain unfavorable parts of this treatment regimen and even after successful treatment, a significant proportion of patients experience recurrence [21]. Consequently, novel therapeutic options are needed to improve the success rate of pulmonary disease associated with MAC infection.

The possibility of multidrug resistance and the safety of available treatment options prompts the need for novel treatment modalities against MAC infection. Emerging evidence suggests antimicrobial peptides (AMPs), including cell-penetrating peptide [R4W4], may confer a therapeutic benefit against mycobacterium infections [30,31]. [R4W4] is cyclic in structure and contains four arginine and four tryptophan residues. Cyclic peptides are ring structures that can be found naturally or synthesized. They have amphiphilic characteristics, allowing them to interact with the cell wall. The cationic parts of the peptides interact with the negatively charged heads of cell membrane lipids, which is furthermore followed by an interaction between the hydrophobic portion and cell wall lipids, which can rupture the cell wall [32]. Depending on the nature of the peptides, they can provide a therapeutic function by acting as agonists, antagonists, RNA-binding molecules, and enzyme inhibitors, along with others. Additionally, arginine and tryptophan are among select amino acids that have demonstrated their ability to increase the activity of antimicrobial peptides [33,34]. Some advantages of using peptides as part of the treatment regimen are their lower risk of toxicity as they do not accumulate in organs, their proteolytic degradation, which yields harmless amino acids, and their larger size that allows them to interact with specific targets only. However, peptides can also have limitations, such as an injectable route of administration, due to poor oral absorption, poor penetration of cell membranes, and their rapid metabolism, which shorten their effect duration [32].

Previous studies testing a variety of cyclic peptides found that [R4W4] demonstrated a high degree of potency against infections, such as methicillin-resistant *Staphylococcus aureus* and *Escherichia coli* [35]. Additionally, [R4W4] showed additive effects when added alongside levofloxacin against selected Gram-positive and Gram-negative bacteria, such as *Klebsiella pneumonia* and *Pseudomonas aeruginosa* [36]. [R4W4] has also been demonstrated to show efficacy against acid-fast bacteria, such as *Mycobacterium tuberculosis (M. tb)*, when used in conjunction with isoniazid or pyrazinamide in *M. tb*-infected peripheral blood mononuclear cells derived from healthy patients [37]. The demonstrated potency of [R4W4] against various pathogens leads us to believe that [R4W4] could be a potent treatment method against *M. avium*. In this study, we aim to evaluate the direct antimycobacterial effects that [R4W4] may possess against *M. avium* culture. We also aimed to determine whether [R4W4] had additive effects when added alongside MAC first-line antibiotics, such as azithromycin and rifampicin.

## 2. Results

### 2.1. M. avium Treated with Cyclic Peptide [R4W4] and Linear Peptide (R4W4)

**Figure 1:**
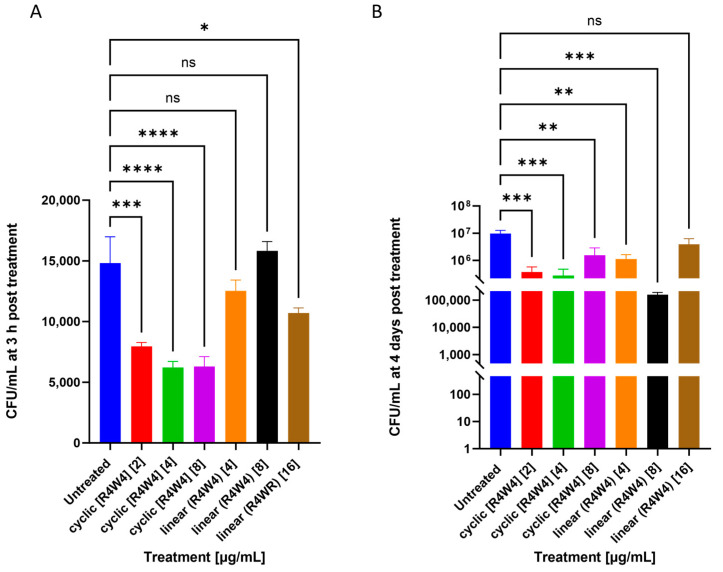
CFU counts of *M. avium* culture treated with cyclic [R4W4] or linear peptide (R4W4). (**A**) CFU/mL of *M. avium* treated with 2, 4, and 8 µg/mL cyclic [R4W4] and 4, 8, and 16 µg/mL linear (R4W4) at 3 h post-treatment; (**B**) CFU/mL of *M. avium* treated with 2, 4, and 8 µg/mL cyclic [R4W4] and 4, 8, and 16 µg/mL linear (R4W4) 4 days post-treatment. *M. avium* survival was tracked by incubating treated bacteria at 37 °C and terminating at 3 h, 4 days, and 8 days post-treatment. Concentration of compound denoted within brackets ([]). GraphPad Prism Software version 9.5.1 was utilized for analysis. Statistical analysis was performed using ANOVA. *p*-values are indicated at the top of each graph, and <0.05 (*), <0.01 (**), and <0.001 (***), <0.0001 (****) were considered significant. Nonsignificant *p*-values are indicated as ns.

We aimed to assess if cyclic [R4W4] exhibited antimicrobial effects against *M. avium.* Cyclic peptide [R4W4] showed significant potency in lowering the number of bacterial colonies at all three minimum inhibitory concentrations when compared with the untreated control group 3 h post-treatment (Figure 1A). We also assessed if the [R4W4] cyclic structure contributed to its function by treating *M. avium* culture with its linear analog, linear (R4W4). The effects of linear peptide (R4W4) were nonsignificant when added at 4 and 8 µg/mL concentrations 3 h post-infection. However, linear peptide (R4W4) showed a significant reduction in bacterial colonies when added at a 16 µg/mL concentration 3 h post-infection. At 4 days post-treatment, both cyclic peptide [R4W4] and linear peptide (R4W4) showed a significant reduction in bacterial colonies when compared with the untreated control group. The highest concentration of linear peptide (R4W4) did not show a significant reduction in bacterial colonies compared with the untreated control 4 days post-infection (Figure 1B).

### 2.2. M. avium Treated with Azithromycin and Cyclic Peptide [R4W4]

**Figure 2:**
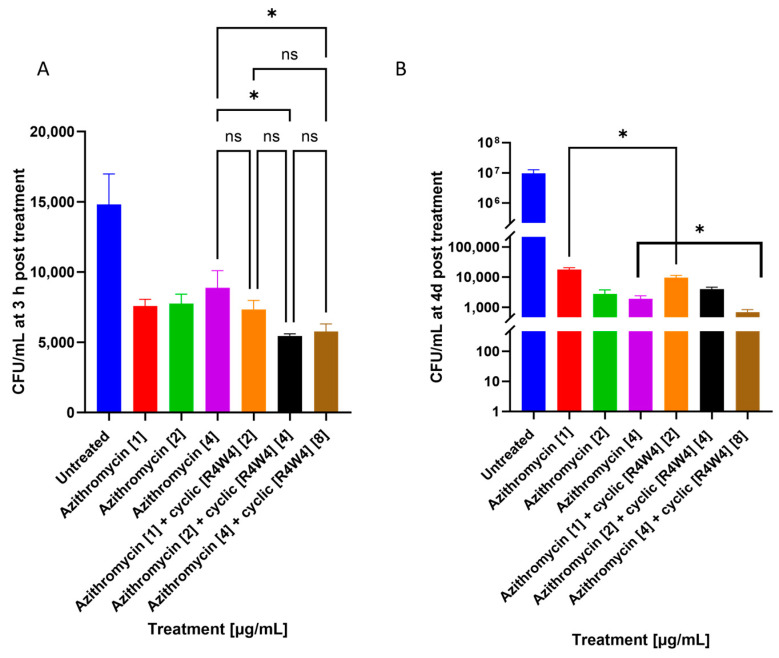
CFU counts of *M. avium* culture without treatment (untreated), azithromycin treatment, or azithromycin–cyclic [R4W4] combination treatment. (**A**) CFU/mL of *M. avium* treated with 1, 2, and 4 µg/mL azithromycin and combination treatment of 1, 2, and 4 µg/mL azithromycin with 2, 4, and 8 µg/mL cyclic [R4W4], respectively, at 3 h post-treatment; (**B**) CFU/mL *M. avium* treated with 1, 2, and 4 µg/mL azithromycin and combination treatment of 1, 2, and 4 µg/mL azithromycin with 2, 4, and 8 µg/mL cyclic [R4W4], respectively, 4 days post-treatment. *Mycobacterium avium* survival was tracked by incubating treated bacteria at 37 °C and terminating at 3 h and 4 days post-treatment. Concentration of compound denoted within brackets ([]). GraphPad Prism Software was utilized for analysis. Statistical analysis was performed using one-way ANOVA. *p*-values are indicated at the top of each graph, and *p*-value < 0.05 (*) were considered significant. Nonsignificant *p*-values are indicated as ns.

Cyclic peptide [R4W4] at a 2 micrograms/mL concentration showed significant additive effects lowering bacterial growth when added with azithromycin at 3 h and 4 days post-treatment. [R4W4] showed additive effects at 8 µg/mL when added with azithromycin at 4 days post-treatment, as shown in Figure 2.

### 2.3. M. avium Treated with Rifampicin and Cyclic Peptide [R4W4]

**Figure 3:**
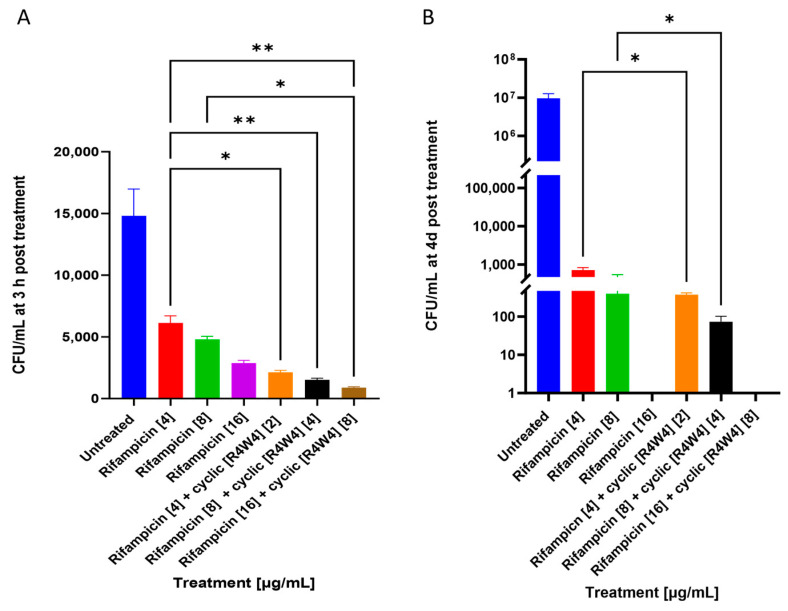
CFU counts of *M. avium* culture without treatment (untreated), rifampicin treatment, or rifampicin–cyclic [R4W4] combination treatment. (**A**) CFU/mL of *M. avium* treated with 4, 8, and 16 µg/mL rifampicin treatment and combination treatment of 4, 8, and 16 µg/mL rifampicin with 2, 4, and 8 µg/mL cyclic [R4W4], respectively, at 3 h post-treatment; (**B**) CFU/mL *M. avium* treated with 4, 8, and 16 µg/mL rifampicin treatment and combination treatment of 4, 8, and 16 µg/mL rifampicin with 2, 4, and 8 µg/mL cyclic [R4W4], respectively, 4 days post-treatment. *Mycobacterium avium* survival was tracked by incubating treated bacteria at 37 °C and terminating at 3 h, 4 days, and 8 days post-treatment. Concentration of compound denoted within brackets ([]) GraphPad Prism Software was utilized for analysis. Statistical analysis was performed using ANOVA. *p*-values are indicated at the top of each graph, and <0.05 (*), <0.01 (**) were considered significant. Nonsignificant *p*-values are indicated as ns.

At 3 h post-treatment, cyclic peptide [R4W4] showed additive effects when added with rifampicin at all three minimum inhibitory concentrations. Cyclic peptide [R4W4] showed additive effects in lowering bacterial growth at 2 and 4 µg/mL concentrations when added with rifampicin at 4 and 8 µg/mL, respectively, at 4 days post-treatment, as shown in Figure 3.

## 3. Discussion

Cyclic peptides are a class of molecules that have been increasingly investigated in the treatment of multidrug-resistant bacterial infections. Several cyclic peptides have been described to elicit bactericidal and bacteriostatic effects against mycobacterium strains, such as *M. tb*. Ecumicin is a macrocyclic tridecapeptide that exerts its antimycobacterial effect by stimulating the ATPase activity of mycobacterial ClpC1 and inhibiting the proteolytic activity of the ClpC1/ClpP/ClpP2 complex [38,39]. Cyclomarin A is another cycloheptapeptide protease inhibitor that interacts with the N-terminal domain of ClpC1 to inhibit mycobacterial growth [40]. Lassomycin binds to a highly acidic region of the ClpC1 ATPase complex [41]. Our laboratory has previously demonstrated that novel cyclic peptide [R4W4] is efficacious against *M. tb* and significantly reduces *M. tb* survival when used in combination with other first-line antibiotics, isoniazid (INH) and pyrazinamide (PZA), in a human granuloma model using peripheral blood mononuclear cells derived from healthy human patients [37].

There has yet to be an exploration of the effects of cyclic peptide [R4W4] on the survival of nontuberculosis mycobacterium (NTM), such as *M. avium*. *M. avium* is the most common causative agent of NTM pulmonary infection in humans. First-line antibiotics against pulmonary *M. avium* include a regimen of azithromycin and rifampicin [42]. Azithromycin is a macrolide that inhibits protein synthesis by binding to the 50S ribosomal subunit [43]. Rifampicin is a first-line antibiotic against *M. avium* and exerts its bactericidal activity by inhibiting RNA synthesis by binding to the bacterial DNA-dependent RNA polymerase [44].

This study aimed to determine the potency of cyclic peptide [R4W4] against *M. avium* infection, both alone and in the presence of azithromycin and rifampicin. Our study showed that cyclic peptide [R4W4] was significantly more potent at lowering bacterial growth when compared with its linear counterpart, (R4W4). Cyclic peptide [R4W4] and linear peptide (R4W4) were added to a bacterial cell culture using the minimum inhibitory concentrations (MICs). The MIC values for cyclic peptide [R4W4] were selected based on previous studies against MRSA [35,45]. The MIC values for rifampicin and azithromycin were selected based on previous bacterial studies [46,47]. We observed that both cyclic and linear [R4W4] can significantly reduce *M. avium* growth 4 days post-infection, though all three concentrations of cyclic peptide [R4W4] were significantly more potent in lowering the growth of *M. avium* when compared with linear peptide (R4W4) for both 3 h and 4 days post-infection (Figure 1A). These findings indicate that the amino acid residues on [R4W4] are sufficient to exert its antibacterial effects, though its cyclical structure contributes to its enhanced efficacy against *M. avium*. These findings are consistent with previous studies testing both configurations of this peptide [48]. Interestingly, the efficacy of cyclic peptide reduces at 8 ug/mL and that of linear peptide reduces at 16 ug/mL 4 days post-infection. The explanation for this effect is unclear. The bacterial rebound is possibly attributable to the compound reaching its maximum efficacy at these concentrations, though further studies are needed to confirm this.

**Figure A1:**
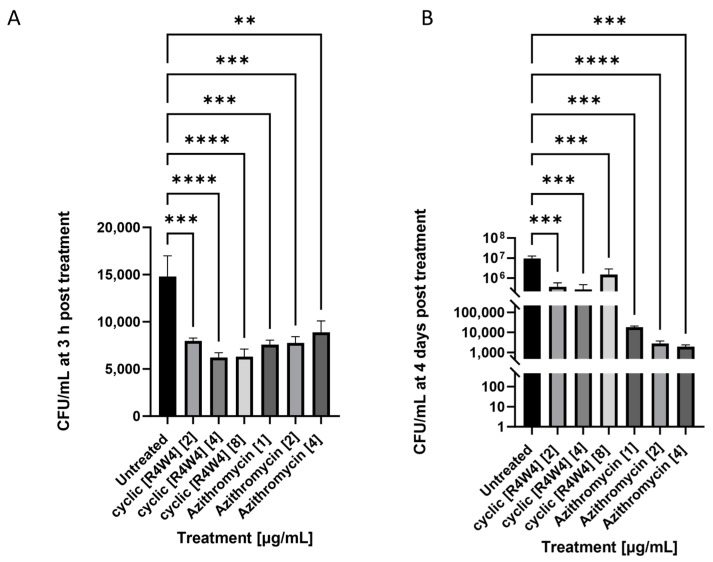
CFU counts of *M. avium* culture without treatment (untreated), cyclic [R4W4], or azithromycin treatment. (**A**) CFU/mL of untreated *M. avium, M. avium* treated with 2, 4, and 8 µg/mL cyclic [R4W4], or 1, 2, and 4 µg/mL rifampicin treatment, respectively, at 3 h post-treatment; (**B**) CFU/mL of untreated *M. avium, M. avium* treated with 2, 4, and 8 µg/mL cyclic [R4W4], or 1, 2, and 4 µg/mL azithromycin treatment, respectively, 4 days post-treatment. *Mycobacterium avium* survival was tracked by incubating treated bacteria at 37 °C and terminating at 3 h, 4 days, and 8 days post-treatment. GraphPad Prism Software was utilized for analysis. Concentration of compound denoted within brackets ([]). Statistical analysis was performed using one-way ANOVA. *p*-values are indicated at the top of each graph, and *p-*value < 0.01 (**), <0.001 (***), and <0.0001 (****) were considered significant.

**Figure A2:**
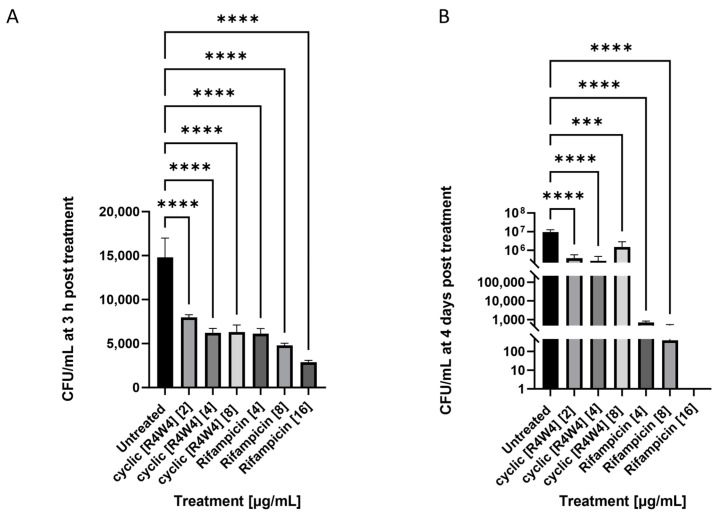
CFU counts of *M. avium* culture without treatment (untreated), cyclic [R4W4], or rifampicin treatment. (**A**) CFU/mL of untreated *M. avium, M. avium* treated with 2, 4, and 8 µg/mL cyclic [R4W4], or 4, 8, and 16 µg/mL rifampicin treatment, respectively, at 3 h post-treatment; (**B**) CFU/mL of untreated *M. avium, M. avium* treated with 2, 4, and 8 µg/mL cyclic [R4W4], or 4, 8, and 16 µg/mL rifampicin treatment, respectively, at 3 h post-treatment and 4 days post-treatment. *Mycobacterium avium* survival was tracked by incubating treated bacteria at 37 °C and terminating at 3 h, 4 days, and 8 days post-treatment. Concentration of compound denoted within brackets ([]). GraphPad Prism Software was utilized for analysis. Statistical analysis was performed using ANOVA. *p*-values are indicated at the top of each graph, and *p-*value < 0.001 (***), and <0.0001 (****) were considered significant.

Our study also demonstrated the additive effects of cyclic peptide [R4W4] when added along with first-line antibiotics, such as azithromycin and rifampicin. Previous reports indicate that the structure of cyclic peptides allows them to have higher receptor sensitivity. Additionally, some cyclic peptides were found to better penetrate cell walls compared with linear peptides [32]. Cyclic [R4W4] has been proposed to exert its antibacterial effect by increasing the permeability of the bacterial cell membrane [35]. We first compared CFU data between cyclic [R4W4]-treated *M. avium* cultures and singular azithromycin and rifampicin treatment. We observed that cyclic [R4W4] alone had comparable antimycobacterial effects to both azithromycin and rifampicin 3 h post-infection (Figure A1A and Figure A2A). However, azithromycin and rifampicin both demonstrated higher reductions in *M. avium* CFU 4 days post-infection compared with cyclic [R4W4] alone (Figure A1B and Figure A2B), suggesting that cyclic [R4W4] alone is not as efficacious as first-line antibiotics at this time point. Cyclic peptide, azithromycin, and rifampicin all significantly reduced *M. avium* CFU compared with untreated controls at both 3 h and 4 days post-infection (Figure A1 and Figure A2). We hypothesize that additive effects will occur when cyclic [R4W4] is added along with either azithromycin or rifampicin, potentially due to cyclic peptide-enhanced cellular uptake of antibiotic treatment.

We found that cyclic peptide [R4W4] demonstrated additive effects when added along with both azithromycin and rifampicin. The use of combination therapy can provide beneficial effects, such as broader antibiotic coverage, lower dosage, shorter duration of treatment, and lower risk of resistance development [49]. The concentrations found to be most potent in lowering the growth of *M. avium* were 2 and 8 micrograms/mL for azithromycin and 2 and 4 micrograms/mL for rifampicin. Both rifampicin and azithromycin are potent antibiotics against a variety of pathogens, but their potency can be enhanced using cyclic peptide [R4W4].

Studies using these treatment categories have not been widely tested against *M. avium* infections. However, these results were anticipated due to the findings from previous studies on MRSA and *M. tb* infections. A previous study found that this cyclic peptide [R4W4] showed enhanced inhibitory effects against *M. tb* infection when added alongside first-line antibiotics, such as tetracycline [37]. Studies using MRSA found that the combination of levofloxacin and [R4W4] demonstrated enhanced killing, which is consistent with our findings demonstrating additive effects of [R4W4] when added along with first-line antibiotics [36].

The findings of our study establish the potency of [R4W4] in lowering the survivability of *M. avium* infection using bacterial cell culture studies. These findings highlight the potential for [R4W4] and permit further infection studies to test potency using different models. The findings of this study are presented with limitations. The *M. avium* strain (*Mycobacterium avium* subsp. *avium*) used in this study was isolated from the liver of a *M. avium-*infected hen, potentially limiting the applicability of the results on *M. avium* strains found in humans [50]. Thus, we recommend further cyclic [R4W4] studies to be performed on *M. avium* subsp. *hominissuis*, the isolate typically found in humans, to confirm the efficacy of cyclic [R4W4] as a treatment modality for *M. avium* complex disease in humans [51]. Additionally, while this study was limited to testing on the bacterial cells directly, a model should be utilized to test the potency of these treatments against *M. avium* in macrophages using methods similar to those in previously published studies [52]. Macrophages play a crucial role in the innate response against controlling *M. avium* infection; however, *M. avium* species have developed multiple mechanisms to evade host killing [53]. As a result, monitoring the activation of macrophages in response to certain treatment regimens can provide insight into how to effectively contain *M. avium* infection. Additionally, using a THP-1-derived macrophage model, we would be able to determine the potency of [R4W4], both alone and in the presence of first-line antibiotics, against intracellular *M. avium* infection. In a study on macrophage–pathogen interactions in zebrafish models, various methods to detect macrophage functions were utilized, such as reactive oxygen species (ROS) and reactive nitrogen species (RNS) response levels, calcium effluxes, apoptosis, and ATP usage [54]. Such methods can allow us to measure the effectiveness of cyclic peptides in containing *M. avium* infection by measuring the activation of macrophages.

In addition to macrophages, future directions for these findings include testing the potency of cyclic R4W4 and combination treatment during an active pulmonary MAC infection in a murine model. C57BL/6, Balb/c, nude, and beige mice have been traditionally utilized for *M. avium* infection studies [55]. However, recently, C3HeB/Fej mice have exhibited necrotic foci during granuloma formation like those observed in humans and not observed in mice, serving as a promising model to evaluate the efficacy of cyclic [R4W4] combination treatment during MAC infection during an active pulmonary MAC infection [55,56]. While cytotoxicity doses have been reported in vitro, randomized placebo-controlled clinical trials (RCTs) in healthy human subjects are needed to assess compound safety and tolerability. Once safety is established, RCTs involving patients with active pulmonary MAC infection are warranted to confirm cyclic [R4W4] as an adjunctive treatment against human MAC infection. This would allow us to work toward implementing these findings in treating immunocompromised humans with *M. avium* infection. However, safety, dosage volumes, and administration method alterations would need to be considered.

## 4. Materials and Methods

### 4.1. Bacterial Processing and Preparation

A laboratory strain of *Mycobacterium avium* (*M. avium)* derived from ATCC 25291™ from KWIKSTIK™ was used for all experiments. *M. avium* was cultured in 7H9 media supplemented with albumin dextrose complex (Hi Media, Santa Maria, CA, USA) and incubated at 37 °C until reaching the logarithmic growth phase at an optical density of 0.5 to 0.8 at A600. *M. avium* cultures were processed to disaggregate bacterial clumps and create a single-cell suspension. Briefly, harvested *M. avium* was centrifuged and washed with 1X phosphate-buffered saline (PBS). Washed *M. avium* was vortexed with 3 mm sterile glass beads at 3 min intervals to disaggregate bacterial clumps. The vortexed bacterial solution was filtered using a 5 µm to eliminate any remaining bacterial aggregations. Processed *M. avium* was serially diluted, plated on 7H11, and incubated at 37 °C to enumerate bacteria in the processed stock. Aliquots of processed stock were stored in individual tubes and stored in a −80 °C freezer until use. All procedures were conducted aseptically in a Class II biochemical safety cabinet.

### 4.2. Bacterial Cell Culture, Antibiotic Treatment, and CFU Counts

To assess the efficacy of antimicrobial peptides and antibiotics on *M. avium* growth, processed *M. avium* cultures (10^5^ CFU/mL) were seeded and cultivated in a 24-well tissue culture plate containing 7H9 and treated with sham sterile PBS control or supplemented with varying concentrations of antibiotics according to the published minimum inhibitory concentrations (MIC) of each antimicrobial agent. Cyclic R4W4 (2 µg/mL, 4 µg/mL, and 8 µg/mL), linear R4W4 (4 µg/mL, 8 µg/mL, and 16 µg/mL), azithromycin (1 µg/mL, 2 µg/mL, and 4 µg/mL), or rifampicin (4 µg/mL, 8 µg/mL, and 16 µg/mL) were supplemented alone or in combination at their corresponding MICs. Treatments were added to their respective wells upon initial infection (Day 0), and 3 days post-*M. avium* infection. Each treatment category was cultured in triplicate and incubated at 37 °C with 5% CO_2_. A study that measured the generation time, or doubling time, of *M. avium* subsp. *paratuberculosis* showed a slow-growing microorganism with a >24 h generation time [57]. To enumerate antibiotic-treated bacterial cultures, small volumes from each well were collected 3 h and 4 days post-infection. Small culture volumes were serially diluted, plated onto MiddleBrook 7H11 Agar Medium in duplicate, and incubated at 37 °C for 11 days. Following incubation, *M. avium* colonies were counted and recorded. All steps were completed aseptically in a Class II biochemical safety cabinet.

### 4.3. Statistical Analysis

Statistical analysis was performed using GraphPad Prism Software. Statistical analysis between treatment categories was performed using one-way ANOVA. Data are reported as the mean ± standard error of the mean. Asterisks between comparison groups indicate *p*-values that are statistically significant. Calculated *p*-values of <0.05 (*), <0.01 (**), <0.001 (***), and <0.0001 (****) were considered statistically significant.

## Data Availability

The data presented in this study are available on request from the corresponding author.
